# A Single-Cell Transcriptome Atlas of the Human Retinal Pigment Epithelium

**DOI:** 10.3389/fcell.2021.802457

**Published:** 2021-12-17

**Authors:** Zongren Xu, Xingyun Liao, Na Li, Hongxiu Zhou, Hong Li, Qi Zhang, Ke Hu, Peizeng Yang, Shengping Hou

**Affiliations:** ^1^ The First Affiliated Hospital of Chongqing Medical University, Chongqing, China; ^2^ Chongqing Key Laboratory of Ophthalmology, Chongqing, China; ^3^ Chongqing Eye Institute, Chongqing, China; ^4^ Chongqing Branch of National Clinical Research Center for Ocular Diseases, Chongqing, China; ^5^ College of Basic Medicine, Chongqing Medical University, Chongqing, China

**Keywords:** retina, HRPE, macula, periphery, single-cell RNA sequencing

## Abstract

Human retinal pigment epithelium cells are arranged in a monolayer that plays an important supporting role in the retina. Although the heterogeneity of specific retinal cells has been well studied, the diversity of hRPE cells has not been reported. Here, we performed a single-cell RNA sequencing on 9,302 hRPE cells from three donors and profiled a transcriptome atlas. Our results identified two subpopulations that exhibit substantial differences in gene expression patterns and functions. One of the clusters specifically expressed *ID3*, a macular retinal pigment epithelium marker. The other cluster highly expressed *CRYAB*, a peripheral RPE marker. Our results also showed that the genes associated with oxidative stress and endoplasmic reticulum stress were more enriched in the macular RPE. The genes related to light perception, oxidative stress and lipid metabolism were more enriched in the peripheral RPE. Additionally, we provided a map of disease-related genes in the hRPE and highlighted the importance of the macular RPE and peripheral RPE clusters P4 and P6 as potential therapeutic targets for retinal diseases. Our study provides a transcriptional landscape for the human retinal pigment epithelium that is critical to understanding retinal biology and disease.

## Introduction

Generally, the retina is a complex structure and contains 10 layers of tissue that are responsible for detecting and converting light into neurochemical information that is ultimately transmitted to the brain, resulting in vision. Near the posterior pole of the human and primate retina is a small shallow funnel-shaped depression 2.5–3 mm in diameter known as the yellow spot or macula. The central depression of the macula is called the fovea, where the retina is the thinnest and has only two layers of cells: the retinal pigment epithelium (RPE) and cone cells. Cone cells and bipolar cells are arranged one-to-one in the fovea, so the fovea, which is the most sensitive and accurate region in the retina, provides a sharp and clear image of central vision. Many ocular diseases that cause blindness, such as age-related macular degeneration (AMD), mainly affect this area, indicating that it is of great significance to study the cellular function of the retina and, particularly, the macula.

The visual formation process requires many types of neurons and supporting cell types. Among these neurons and cells, photoreceptor (PR) cells (rods and cones) convert light into an electrical signal that is then transferred to interneurons, including horizontal cells (HCs), bipolar cells (BCs), and amacrine cells (ACs). Interneurons deliver information to retinal ganglion cells (RGCs) and then input it into the brain. In addition, RPE cells, astrocytes, Müller glia and microglial cells mainly support the metabolism of the retina and play an important role in homeostasis of the retina.

Some retinal cell types have been studied for their gene expression patterns and gene functions by bulk sequencing, even at the single-cell level. Recent research found that RGCs are divided into 40 cell types by single-cell RNA sequencing (scRNA-seq) (Rheaume et al., 2018). Additionally, rods and cones have exhibited heterogeneous subpopulations (Yan et al., 2020). Many studies have reported retinal cell subtypes, including RGCs, rods, cones and other nonneuronal cells, in humans and other primates as determined by scRNA-seq (Shekhar et al., 2016; Rheaume et al., 2018; Lukowski et al., 2019; Menon et al., 2019); however, the heterogeneity and detailed molecular map of human RPE cells has not been well studied.

The RPE is a monolayer tissue layer that is fundamentally important for retinal development and function. The RPE also plays critical roles in supporting the retina, including transepithelial transport, phagocytosis, blood-retina barrier function, metabolism, oxidative stress (OS), growth factor secretion, visual cycle processes (Strauss, 2005) and retinal integrity and viability maintenance (Boulton and Dayhaw-Barker, 2001; Bharti et al., 2011). An increasing number of studies have shown that RPE dysfunction may lead to multiple retinal degenerative diseases, such as AMD, Stargardt’s macular dystrophy (SMD), best vitelliform macular dystrophy (BVMD) and proliferative vitreoretinopathy (PVR). Although a number of studies have focused on deriving RPE cells from various stem cell sources or cell lines and have even focused on the functions of RPE cells, precise cell-type division has not been examined in human RPE (hRPE) cells. Therefore, it is necessary to explore the heterogeneity and molecular map of hRPE cells to elucidate the mechanism of hRPE-related retinal diseases and discover more treatments for these diseases. In view of the important roles of hRPE cells, we therefore performed scRNA-seq from three human donor eyes to study the heterogeneity and gene expression in RPE tissues.

## Results

### The Preparation of hRPE Samples and Generation of a Single-Cell Transcriptome Atlas

**FIGURE 1:**
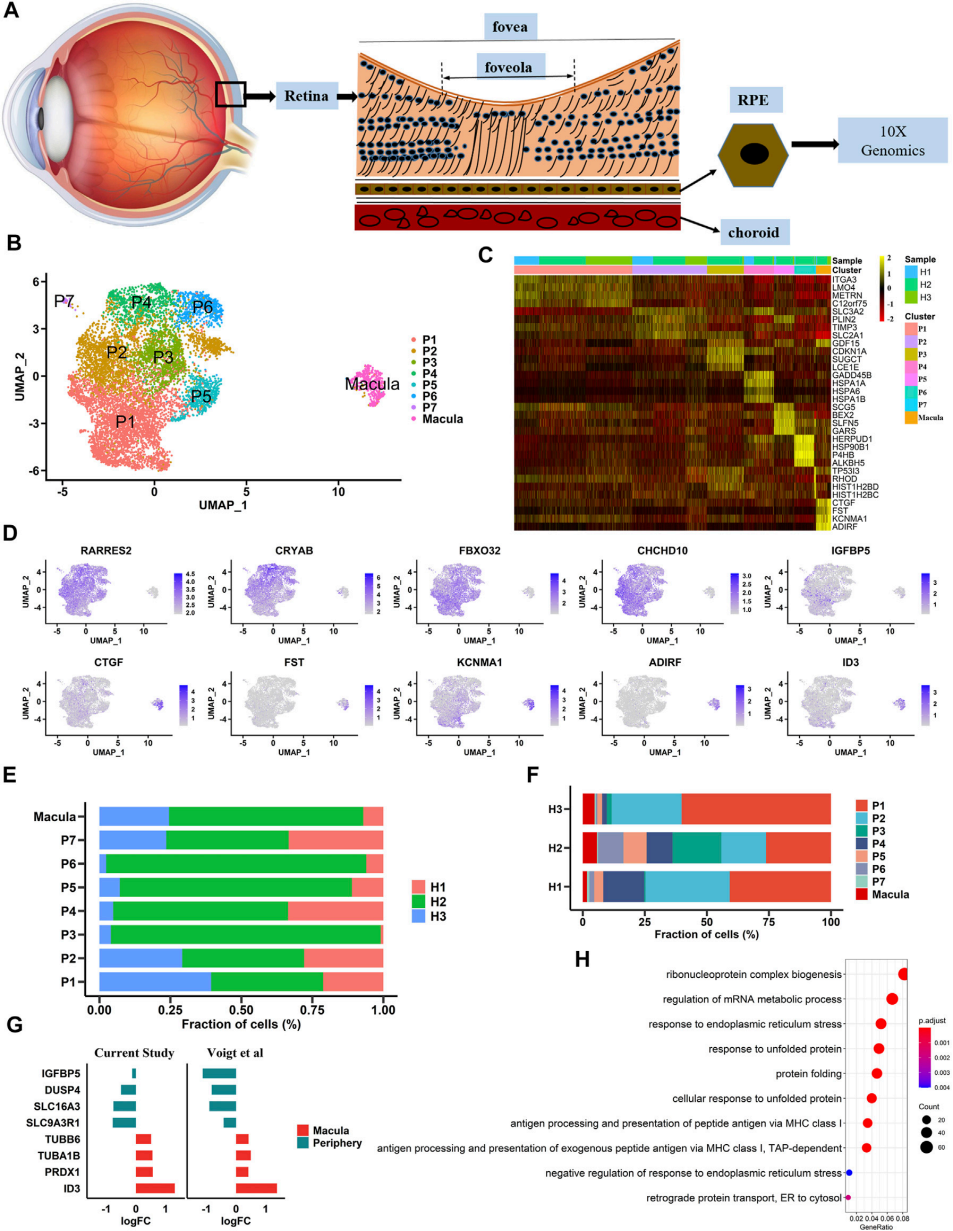
Single-cell RNA-seq transcriptome profiling of hRPE **(A).** A schematic diagram of the extracted sample cells is shown. Single cells were extracted from human RPE tissue to complete next-generation sequencing **(B)**. Identification of cell populations. UMAP projection of 9,302 single cells from three samples showed a total of eight cell type populations. Each point is a cell, and different cell types are differentiated by different colors **(C)**. Heatmaps of cell types. Columns represent groups of cells, and rows represent specific, highly expressed differentially expressed genes for each cell type. The heatmap scale represents the normalized expression value **(D)**. Differential gene expression in large and small clusters is shown in the UMAP plot **(E)**. The proportion of each sample in each cluster **(F)**. The proportion of each cluster in each sample **(G)**. The expression patterns of differential genes in our data were consistent with those in previous studies **(H)**. Gene Ontology (GO) terms associated with genes with upregulated expression in the macula cluster.

In this study, three postmortem human adult eyes were obtained after corneal transplantation. As the transcriptome profile of human retinal cells, including HCs, BCs, and microglia, has been reported, we focused solely on building a single-cell transcriptome atlas of hRPE cells with a 10× Genomics Chromium platform (Figure 1A). A total of 10,074 cells were obtained by single-cell sequencing with an average of 200,801 reads and 5,522 median genes per cell. After rigorous quality control and filtering using the Seurat package (version 3.1.5) (Stuart et al., 2019), 9,302 cells were included in the follow-up unsupervised graph clustering approach. The hRPE cell atlases yielding high-quality cell profiles were divided into two populations: one population had 8,863 cells, and the other population had 439 cells (Figures 1B, C and Supplementary Figure S1A-B). Both populations expressed RPE marker genes such as *PAX6* and *BEST1* (Supplementary Figure S1C). Previous transcriptome studies showed that *ID3* and *IGFBP5* were highly expressed in the macular and peripheral regions of hRPE cells, respectively (Voigt et al., 2019). Interestingly, our study also found that specific expression of *ID3* was limited to a small population (439 cells) and that *IGFBP5* was highly enriched in the other population (8,863 cells). Thus, we hypothesized that one cluster was the macular RPE and the other was the peripheral cluster. In addition to the two specifically expressed genes, we found that *CTGF*, *FST*, *KCNMA1,* and *ADIRF* were highly expressed in the macular RPE, and *RARRES2*, *CRYAB*, *FBX O 32*, and *CHCHD10* were specifically expressed in the peripheral RPE (Figure 1D and Supplementary Table S1). In addition, we found two populations with similar ratios in the three human samples (Supplementary Figure S1E–F). We also found that the proportions of these subpopulations were similar among samples (Figures 1E, F). At the same time, we compared the work reported by Voigt and others to reproduce the expression of these differentially expressed genes (Figure 1G). Next, we performed Gene Ontology (GO) pathway enrichment analysis on highly expressed genes in peripheral and macular RPE cells (Figure 1H, Supplementary Figure S1D and Supplementary Table S2). The results showed that there may be significant differences between peripheral RPE cells and macular RPE cells in proton transmembrane transport, endoplasmic reticulum (ER) stress and ATP metabolic processes.

### Profiling of Human Macular RPE Cell Subpopulations

**FIGURE 2:**
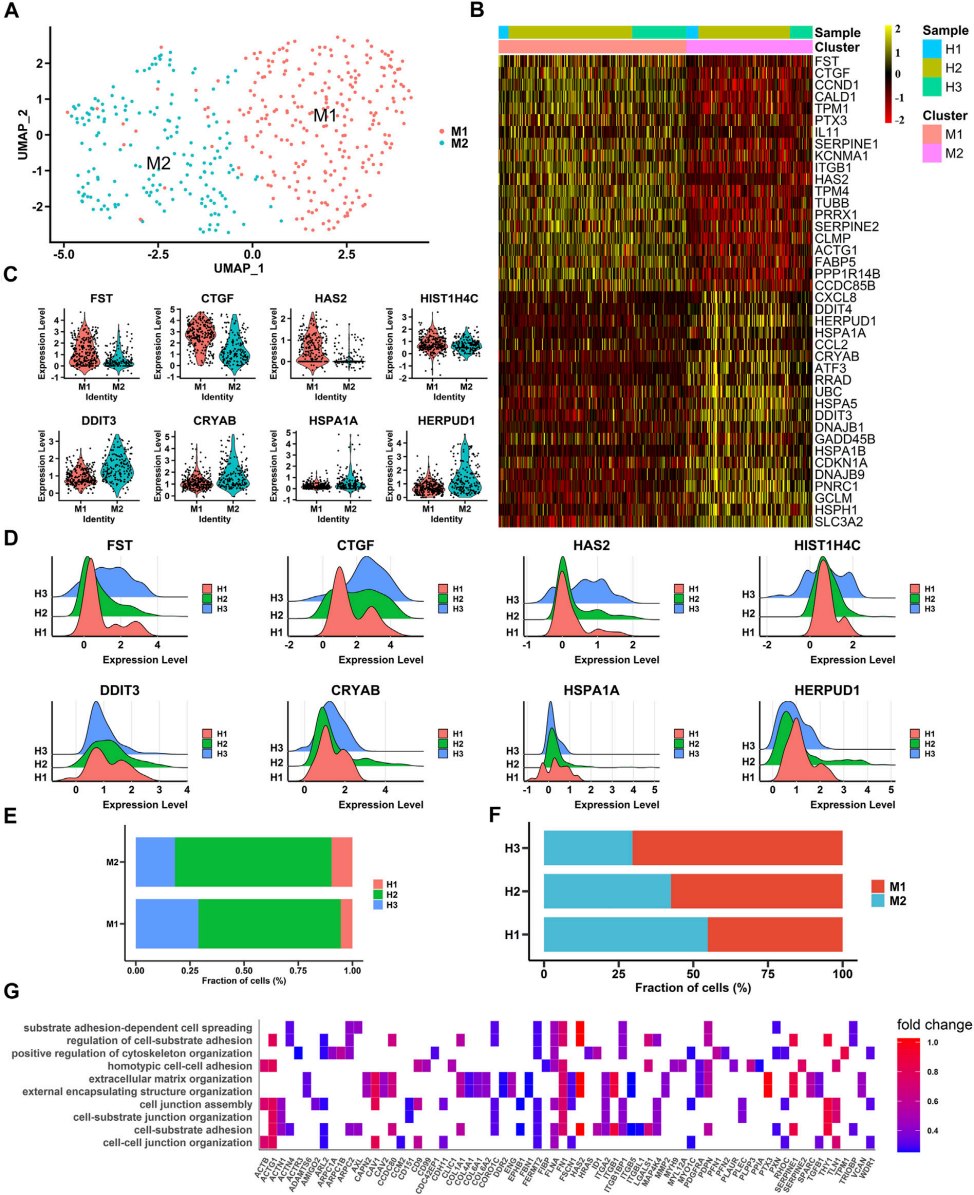
Single-cell RNA-seq transcriptome profiling of macular RPE **(A)**. UMAP plot shows the cell classification of macular RPE cell clusters in three samples **(B).** Heatmap of two macular RPE subpopulations. Columns are divided into two types of cells, rows represent gene names and can be used to identify two subpopulations. The heatmap scale represents the normalized expression value **(C).** Violin plots show the expression of differentially expressed genes, M1 **(top)** and M2 **(bottom)** **(D).** Ridge plots show the expression patterns of differentially expressed genes in three samples. The *X*-axis shows the level of gene expression. M1 **(top)** and M2 **(bottom)** **(E).** The proportion of each sample in each cluster (M1, M2) **(F).** The proportion of each cluster (M1, M2) in each sample **(G).** Gene Ontology (GO) terms associated with genes with upregulated expression in the M1 subpopulation.

To gain insight into the heterogeneity of macular RPE cells, we analyzed the macular RPE cluster and found that it could be divided into two subpopulations (M1 and M2 clusters) according to the gene expression profile (Figures 2A, B). After differential expression analysis, we obtained a total of 585 differentially expressed genes (Supplementary Table S3). These genes were clearly identified in two subpopulations and showed similar expression patterns in all three samples (Figures 2B–D). In addition, the statistical proportions of the two subgroups were very similar (Figures 2E, F). We further performed GO analysis to explore the physiological functions of the two clusters. The results indicated that the M1 cluster was enriched in cell adhesion and cell junctions, and the M2 cluster was predominantly enriched in OS and ER stress in response to illness or trauma (Figure 2G, Supplementary Figure S2 and Supplementary Table S4). The results demonstrated that the macular RPE could also be divided into smaller subpopulations that perform different functions.

### The Subpopulations and Expression Profile of Human Peripheral RPE Cells

**FIGURE 3:**
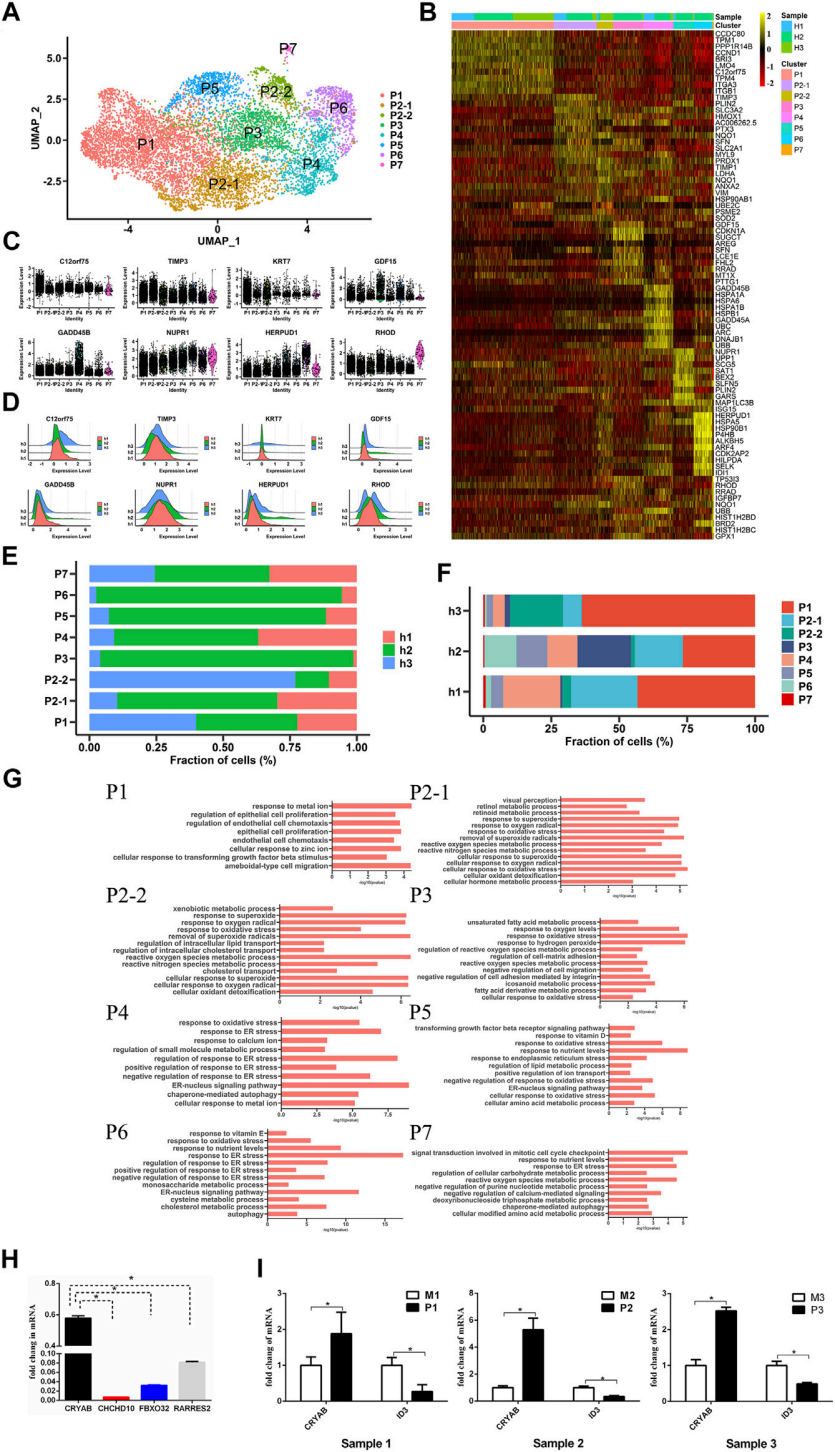
Single-cell RNA-seq transcriptome profiling of peripheral RPE **(A).** UMAP plot shows the cell classification of peripheral RPE cells in three samples **(B).** Heatmap of eight peripheral RPE subpopulations. Columns are divided into eight types of cells, and rows represent gene names and can be used to identify eight subpopulations. The heatmap scale represents the normalized expression value **(C).** Violin plots showing 8 clusters of peripheral RPE-specific genes **(D).** The expression patterns of the differentially expressed genes in the three samples. The *X*-axis shows the level of gene expression **(E).** The proportion of each sample in each cluster (P1, P2-1, P2-2, P3, P4, P5, P6, P7) **(F).** The proportion of each cluster (P1, P2-1, P2-2, P3, P4, P5, P6, P7) in each sample **(G).** Gene Ontology (GO) terms associated with genes with upregulated expression in each peripheral RPE population **(H).** RT-PCR analysis of differentially expressed genes with high specific expression in peripheral RPE. *: *p* < 0.05 **(I).** Verification of peripheral RPE markers and macular RPE markers by RT-PCR.

To study the heterogeneity of peripheral RPE cells, we mapped their atlas and showed that peripheral RPE cells could be classified into eight populations with distinct gene expression (Figures 3A, B). We calculated and attained specific differential genes for each subpopulation and then found similar expression patterns in all three samples (Figures 3B–D and Supplementary Table S5). In addition, the statistical graph also showed the number of three samples in the subpopulations (Figures 3E, F). To further determine the functional differences among the peripheral subpopulations, we profiled the GO analysis results; the findings showed that these subpopulations had functional differences (Figure 3G and Supplementary Table S6). The P1 cluster, the largest peripheral subpopulation, was found to be mainly responsible for extracellular matrix organization, indicating that this cluster played an important role in maintaining the structure and stability of RPE tissue. Intriguingly, the P2 cluster could be divided into P2-1 and P2-2, and these subclusters had obviously different functions. The P2-1 cluster was mainly related to retinol metabolic processes and visual perception. However, the P2-2 cluster was involved in lipid transport. The P3 cluster was associated with OS. In addition, the P5 cluster was associated with nutrient transport. Although the two clusters P4 and P6 had similar functions, which were both related to ER stress, there were differences between them. We observed that the P4 cluster was more strongly associated with growth factors and ions; however, the P6 cluster was related to nutrient transport. The P7 cluster may be associated with cell cycle. In summary, we first profiled the transcriptomes of peripheral RPE cells in the human retina, and the presented data showed the heterogeneity of these peripheral RPE cells.

Our scRNA-seq results showed a low specificity of *IGFBP5* in the data (Figure 1D). Therefore, real-time quantitative polymerase chain reaction (RT-PCR) analysis of some differentially expressed genes was conducted, and the results showed that *CRYAB* expression was significantly higher in peripheral RPE than that of other differentially expressed genes (Figure 3H). Therefore, we determined that *CRYAB* was more specific than *IGFBP5* and could serve as a new marker gene of peripheral RPE. Then, we also verified the peripheral RPE marker gene (*CRYAB*) and macular RPE marker gene (*ID3*) by RT-PCR analysis (Figure 3I).

### Different Gene Expression Patterns Between Macular and Peripheral RPE

**FIGURE 4:**
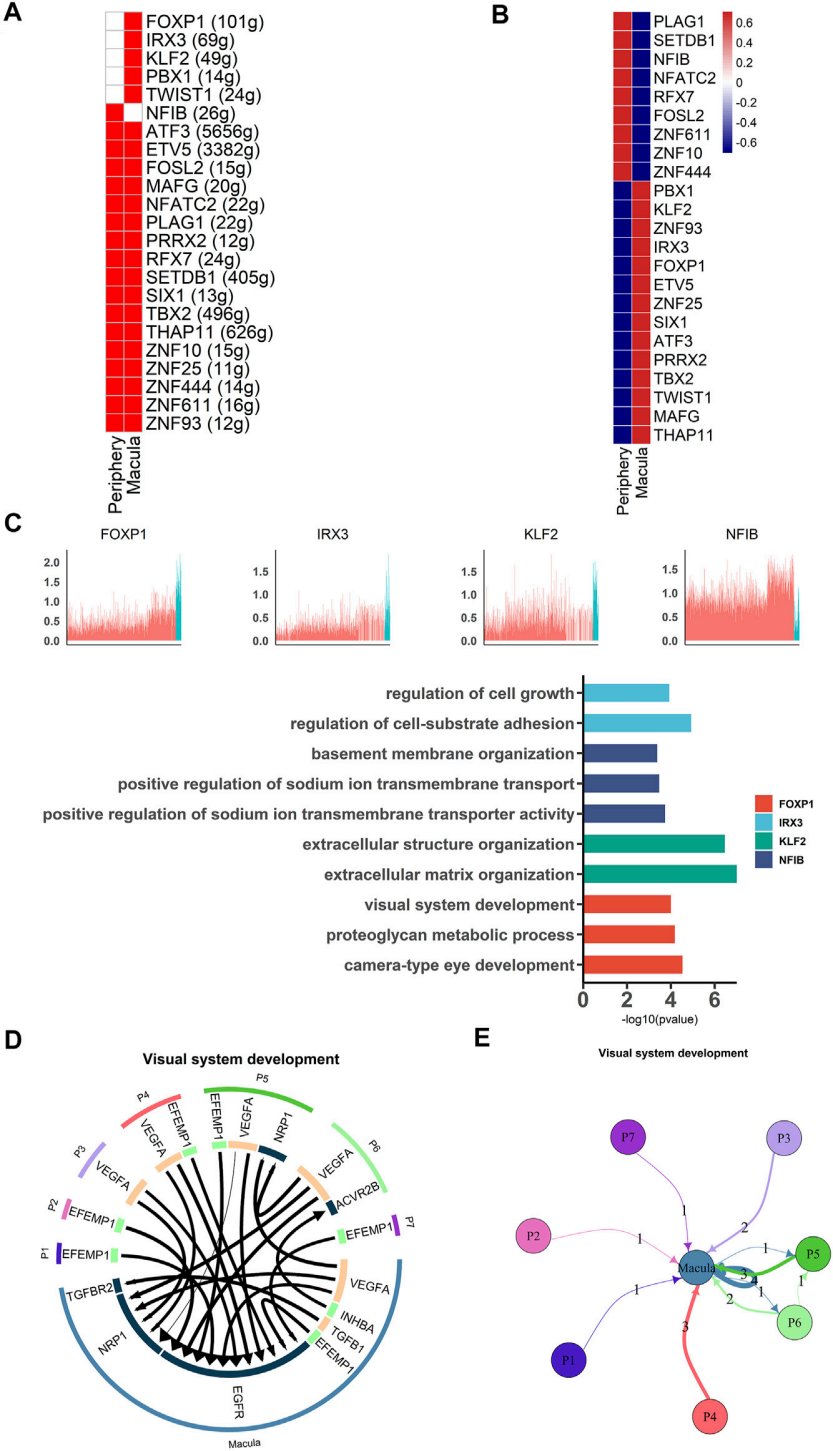
Different gene expression patterns between macular and peripheral RPE **(A).** SCENIC results for the macular RPE and peripheral RPE. Heatmap showing the active and expressed transcription factors in each cell cluster. The states of the transcription factors in each cell class are indicated in red (active) and white (inactive) **(B).** Heatmap shows the average expression of active transcription factors in each cell cluster. The expression level is indicated by the color level; red indicates high expression, and blue indicates low expression **(C).** Expression of specific highly expressed transcription factors and GO functional analysis of their corresponding target genes. The upper part of the figure represents the expression of transcription factors, and the lower part of the figure represents the functional annotation of target genes regulated by transcription factors **(D).** Analysis of cell communication between macular RPE and peripheral RPE. The visual system development of the hRPE among the top 20 ligand-receptor pairs **(E).** Connections between the peripheral RPE subpopulations and the macular RPE cluster of ligand-receptor pairs of the visual development system.

Transcription factors are necessary for RPE development. We therefore used single-cell regulatory network inference and clustering (SCENIC) (Aibar et al., 2017) to analyze the activity of the gene regulatory networks (GRNs) in each cell. Based on a previous study, transcription factors that were active in more than 50% of cells in a particular cell class were retained (Hu et al., 2019). High activities of transcription factors were detected from the data using the AUCell algorithm (Figure 4A, B ). Our results showed that *NFIB* was active and highly expressed in peripheral hRPE cells and that its target genes were involved in basement membrane organization and sodium ion transmembrane transport (Figure 4C). *IRX3, FOXP1, KLF2, TWIST1* and *PBX1* were highly expressed in macular hRPE cells and were considered active transcription factors. Target genes of *IRX3* were related to cell growth and cell-substrate adhesion. Target genes of *KLF2* were involved in extracellular structure organization and matrix organization. Target genes of *FOXP1* were enriched in regions involved in visual system development, proteoglycan metabolic processes and camera-type eye development. Therefore, these results indicate that most of the transcription factors were active in the macular cluster, suggesting that the macular cluster may play an important role in the differentiation of RPE cells during retinal development.

To further investigate the importance of macular clusters, we analyzed the interaction between peripheral and macular populations and then examined receptor-ligand pairs between the subpopulations. We found that 17 of the top 20 receptor-ligand pairs were enriched in the macular RPE, suggesting that the macular RPE cluster was closely involved in visual system development (Figures 4D, E). For example, *EGFR* receptors account for a large proportion of the macular RPE and mainly bind to *VEGFA* and *EFEMP1* (Fernandez-Godino et al., 2015; Mackay et al., 2015). Moreover, *NRP1* is another key receptor of the macular PRE whose signaling pathways are primarily related to angiogenesis and neural development (Raimondi et al., 2016). In summary, our data revealed the expression patterns of transcription factors in the human RPE, as well as the interactions between RPE subpopulations, both of which suggest the importance of the macular RPE in visual development.

### The Development of the hRPE and Its Dynamic Transcriptome Features

**FIGURE 5:**
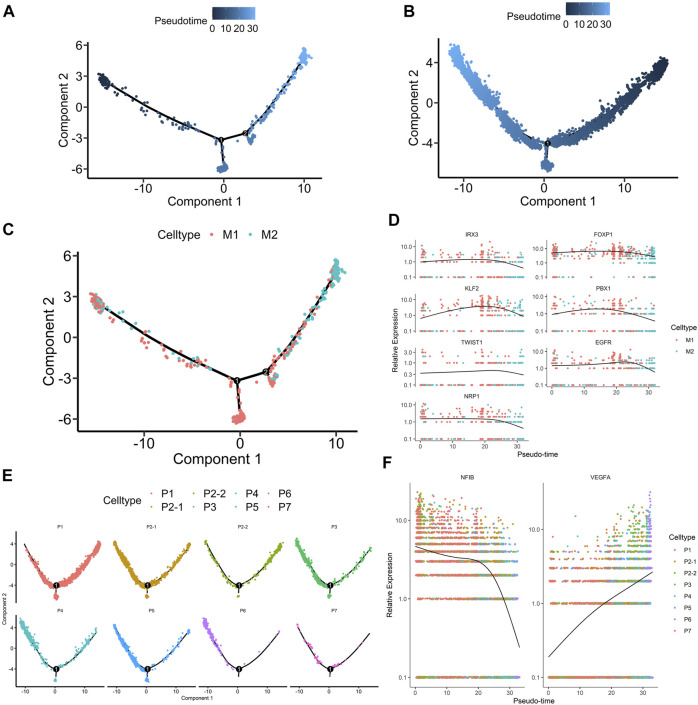
The development of human peripheral RPE and macular RPE **(A-B).** Trajectory analysis of the transition of the hRPE along pseudotime. The shades of color represent the order of the time **(A).** macular RPE **(B).** peripheral RPE **(C).** Trajectory analysis of macular RPE subpopulations over pseudotime. Cell types are distinguished by different colors **(D).** Pseudotime changes in important transcription factors and receptors associated with macular RPE **(E).** Trajectory analysis for peripheral RPE subpopulations along pseudotime. Cell types are distinguished by different colors **(F).** Pseudotime changes in important transcription factors and receptors associated with peripheral RPE.

We performed pseudotemporal analysis to elucidate the transcriptome dynamics of peripheral and macular RPE clusters using the R package Monocle2 (Trapnell et al., 2014). The discriminative dimensionality reduction tree (DDRTree) algorithm was used to determine the pseudodevelopmental time and then to map the developmental trajectory (Figures 5A–C and Figure 5E). It is well known that the anatomical development of the macula is complete at a late stage after being born (Alabduljalil et al., 2019). Therefore, we wanted to investigate the dynamics between the subpopulations of macular RPE and peripheral RPE. In macular RPE, M1 cluster cells were in the early stage of development, and M2 cluster cells were in the late stage of development (Figure 5A and Figure 5C). In peripheral RPE, we also found that these subpopulations had a specific developmental sequence (Figure 5B and Figure 5E).

Further studies also observed that the specific transcription factors of the macular and peripheral RPE, including *IRX3*, *TWIST1*, *FOXP1*, *KLF2*, *PBX1* and *NFIB,* were changed along the pseudotime axis (Figures 5D–F). For example, the expression of *IRX3*, *TWIST1*, *FOXP1*, *KLF2* and *PBX1* was decreased gradually in macular RPE (Figure 5D). The expression of *NFIB* was also decreased gradually in peripheral RPE (Figure 5F). Additionally, we analyzed the changes in receptors that are key for the function of the RPE (Figures 5D–F). The results showed that the expression of EGFR and NRP1 receptors decreased during development in macular RPE (Figure 5D). However, the expression of the *VEGFA* ligand increased gradually in peripheral RPE (Figure 5F).

### Profiling of Specific Expression Patterns of Human Retinal Disease-Associated Genes

**FIGURE 6:**
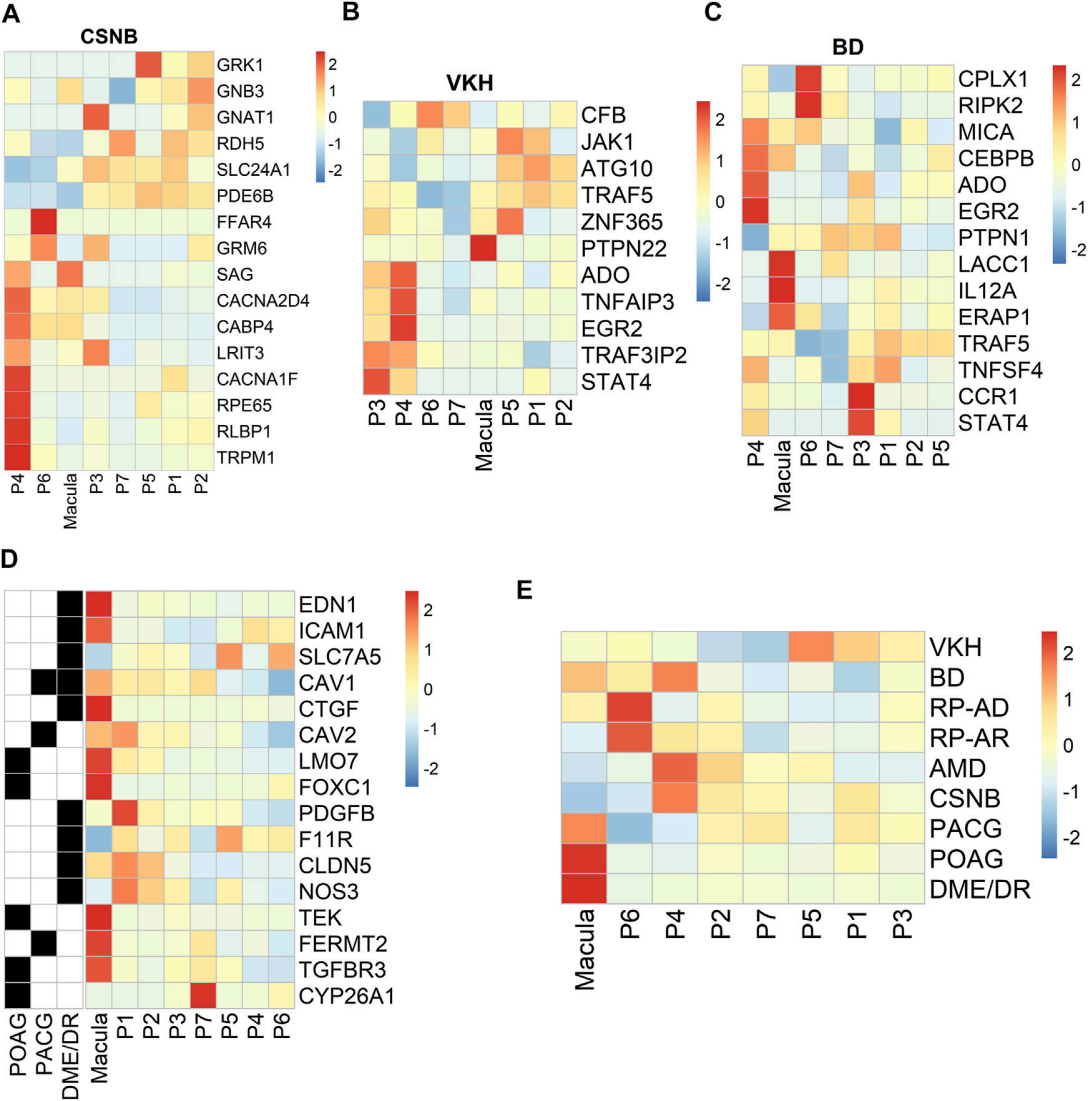
Profiling of specific expression patterns of human retinal disease-associated genes. Aggregated expression of disease-associated genes in macular and peripheral cell types. CSNB, congenital stationary night blindness; BD, Behcet disease; VKH, Vogt-Koyanagi-Harada syndrome. Colorbar: red represents highly expressed and blue represents lowly expressed, scale method is Z-score normalization **(A).** CSNB **(B).** VKH **(C).** BD **(D).** Expression patterns of specific retinal disease-associated genes (rows) by HRPE in the macula and periphery (columns) **(E).** Known or candidate genes for multiple ocular diseases. Ocular diseases are shown in rows, columns are cell clusters identified.

We used the RetNet (https://sph.uth.edu/retnet/) database to identify genes associated with retinal diseases and to determine the cluster distribution of these genes in our data. Studies have reported that *USH2A*, *EYS* and *CRB1* are the top three genes responsible for inherited retinal dystrophy (Huang et al., 2015). In our data, *CRB1* and *EYS* were related to the development of the autosomal recessive disorder retinitis pigmentosa (RP). These genes were mainly expressed in the P3, P4, P5 and P7 clusters. The genes *LRAT*, *RPE65* and *RLBP1* are associated with the visual cycle (Strauss, 2005; Lima de Carvalho et al., 2020). *LRAT* was a susceptible gene of autosomal recessive RP that was highly expressed in the P5 cluster in our data. Our results demonstrated that the *PRPH2*, *PRPF6*, and *IMPG1* genes associated with autosomal dominance of RP were highly expressed in the macular RPE and clusters P6 and P7 (Supplementary Figure S3A, B). *RPE65* and *RLBP1*, which were highly and specifically expressed in the P4 cluster, are susceptible genes contributing to RP and congenital stationary night blindness (CSNB) (Figure 6A). In previous studies, *VTN* and *HTRA1* associated with AMD were highly expressed in PR cells and HCs in the retina, respectively (Fritsche et al., 2016; Peng et al., 2019; Orozco et al., 2020). Interestingly, in our hRPE data, we found that the *VTN* and *HTRA1* genes were highly expressed in clusters P2 and P7 (Supplementary Figure S3C). In addition, other retinal disease-causing genes related to AMD were mainly expressed in clusters P4, P6 and P7, but some susceptibility genes, such as *C2*, *FBLN5*, and *TLR4,* were highly expressed in the macular cluster (Supplementary Figure S3C).

From the genome-wide association study (GWAS) results (Hou et al., 2014; Wang et al., 2019a), we found region-specific expression of genes implicated in human retinal diseases such as Behcet’s disease (BD) and Vogt-Koyanagi-Harada syndrome (VKH). VKH-related genes were mainly highly expressed in clusters P3 and P4, and *PTPN22* was specifically expressed in macular RPE cells (Figure 6B). BD-related genes were mainly highly expressed in P3, P4, P6 and macular RPE clusters (Figure 6C). Next, we analyzed ocular disease-associated gene expression in subpopulations and found that many human retinal diseases primarily affected the macular RPE cluster and some peripheral RPEs, such as clusters P4 and P6 (Figures 6D, E). In summary, these results identify the subpopulation distribution of multiple ocular disease-related genes in the RPE and may provide information for future genetic correlation analysis and disease treatments.

## Discussion

Single-cell sequencing technology is helpful to analyze cell types and gene expression patterns for many complex tissues in detail. Single-cell sequencing of hRPE cells from three adult donor eyes was performed to investigate the heterogeneity of the RPE, including cell classification and functional differences. Our results showed that RPE tissues could be categorized into two clusters, one of which is macular RPE highly expressing *ID3* and the other is peripheral RPE cluster expressing *CRYAB*. A recent study of single-cell sequencing of the RPE found that the expression of *ID3* and *IGFBP5* was enriched in the macular and peripheral RPE, respectively (Voigt et al., 2019). Similarly, our results showed that the hRPE was divided into two clusters and that one cluster had a small number of cells, a total of 439 cells, highly expressing *ID3*. However, we found that *CRYAB* was more specific than *IGFBP5* in peripheral hRPE cells, indicating that *CRYAB* may be firstly identified as a new marker gene of peripheral RPE cells. In addition, we found that the expression of *BEST1* in the macular cluster was lower than that in the peripheral cluster, which was consistent with a previous study (Mullins et al., 2007; Voigt et al., 2019). Furthermore, according to their transcriptome expression profiles, the two clusters could be divided into more elaborate subpopulations, including P1, P2-1, p2-2, P3, P4, P5, P6, and P7 and M1 and M2 clusters. Further studies are needed to elucidate the biological functions of each hRPE subpopulation.

Previous studies have described the transcriptome landscape of the human retinal tissues by scRNA-seq (Hu et al., 2019) and snRNA-seq analysis (Liang et al., 2019). As we did not examine the photoreceptor cells using scRNA-seq analysis, further study was needed to clarify the interaction of the RPE and the retinal cells.

As we all know, the RPE has various functions. Our results showed that OS-related genes were more highly expressed in the macular cluster than in the peripheral cluster, which may imply the stronger ability of the macula to respond to external stimuli and injuries. Although the RPE has metabolic and transport functions, the peripheral cluster of the hRPE was found to be involved in lipid metabolism, while the macular cluster favored metal ion transport. Further studies revealed that the two subpopulations of macular RPE cells performed different functions. One of the populations was related to cell adhesion (M1); however, the other population (M2) highly expressed several genes, such as *HERPUD1*, *HMOX1*, *MDM2*, and *XBP1*, which are related to OS and ER stress. Interestingly, the aforementioned genes were also found to play a crucial role in human macula development and cone functions (Kohl et al., 2015). These aforementioned results strongly indicated the heterogeneity of molecular expression and functions between the macula and the peripheral hRPE.

Transcription factor and intercellular communication analyses on hRPE cells were also performed; the results of these analyses indicated that macular and peripheral hRPE clusters had different expression patterns of transcription factors. Interestingly, EGFR plays an important role in cell growth, proliferation and differentiation (Martín-Bermudo et al., 2015; Malartre, 2016) and was found to be enriched in the macular hRPE cluster but not in the peripheral hRPE cluster. In contrast, several ligands of EGFR were highly expressed in the peripheral hRPE. EGFR has been shown to regulate autophagy and phagocytosis of RPE cells (Muniz-Feliciano et al., 2017) and the proliferation of retinal progenitor cells (Close et al., 2006). Accumulating evidence suggests that macular clusters with high expression of EGFR may have more important functions, such as autophagy, phagocytosis and cell proliferation, in the RPE. Interestingly, *TYRP1* has been reported to be involved in melanin synthesis (Rai et al., 2020), and *RLBP1* is related to the visual cycle (Wen et al., 2016). Our study showed that both of these genes were highly expressed in the peripheral P2-1 cluster (Figure 3G).

In the pseudotemporal analysis, it is evident that these subpopulations had a chronological developmental sequence. In macular RPE, the results combined with the GO analysis results showed that M1 cluster cells grew to M2 cluster cells. In the peripheral RPE, these results suggested that the more complex the function of subpopulations was, the later the developmental time. In addition, these important transcription factors, receptors and ligands may play important roles in early development except *VEGFA*, *and* it is possible that *VEGFA* acts later in the development of RPE cells.

About 200 susceptibility genes related with VKH, BD, AMD, and other human ocular diseases were also analyzed. Although these many genes implicated in ocular diseases were found to be mainly expressed in PRs and nonneuronal cells (Peng et al., 2019), our results showed that some susceptible genes were also expressed in the hRPE subgroups. Additionally, primary open-angle glaucoma (POAG), primary angle-closure glaucoma (PACG), diabetic macular edema and retinopathy (DME/DR), AMD and BD were closely related to the population of the macula or P4 clusters. This evidence suggests that a specific hRPE subpopulation is associated with certain ocular diseases, and more attention to specific cell subsets of the hRPE should be paid when studying disease mechanisms.

It is also worthwhile to point out that some questions need to be further investigated. Many ocular diseases such as AMD and RP are closely related to the macular retina, especially RGCs, cones, and rod cells; however, our results showed that many susceptibility genes associated with these diseases were selectively expressed in a particular macular RPE subpopulation. The pathogenesis of hRPE cell subsets in these diseases needs to be further explored. Additionally, due to the heterogeneity of hRPE cells and the influence of age on their heterogeneity, further studies are needed to clarify the physiological and pathological function of hRPE clusters.

In conclusion, although the RPE is a single-layer epithelium, we constructed the transcriptome landscape of the human RPE, as well as a retinal disease map of the hRPE atlas, and obtained key information about the heterogeneity and specific functions of cell subpopulations in the human RPE; this information is likely to be an important clue for understanding the cellular mechanisms and curing pathological conditions of the hRPE related to ocular diseases.

## Materials and Methods

### Ethics Statement

The present study was approved by the Ethics Committee of the First Affiliated Hospital of Chongqing Medical University, China (Permit Number: 2019-099) and adhered to the tenets of the Declaration of Helsinki. All eye tissue samples used in this study were collected from Chongqing Eye Bank, the First Affiliated Hospital of Chongqing Medical University. Written informed consent was obtained from the patients prior to the study.

### Reagents

All the primers used in this study were synthesized by Shanghai Sangon Biological Engineering Technology and Services. The primers were ID3-F:

5′ GAG​AGG​CAC​TCA​GCT​TAG​CC3’, ID3-R:

5′TCC​TTT​TGT​CGT​TGG​AGA​TGA​C3’; RARRES2-F: 5′GCA​TCA​AAC​TGG​GCT​CTG​AG3′, RARRES2-R: 5′AGG​GAA​GTA​GAA​GCT​GTG​GG3’; CRYAB-F: 5′GGG​AGA​TGT​GAT​TGA​GGT​GC3′, CRYAB-R: 5′TTC​ACA​GTG​AGG​ACC​CCA​TC3’; FBXO32-F: 5′TGT​GGG​TGT​ATC​GGA​TGG​AG3′, FBXO32-R: 5′GAG​TTT​CTT​CCA​CAG​CAG​CC3’; CHCHD10-F: 5′CAG​AGT​GAC​CTG​TCC​CTG​TG3′, and CHCHD10-R: 5′CCT​CCT​CAC​TTC​CAA​TCC​CA3’. Cy3-labeled goat anti-rabbit IgG (H + L) (Beyotime, A0516, 1:500), Cy3-labeled goat anti-mouse IgG (H + L) (Beyotime, A0521, 1:500), Alexa Fluor 488-labeled goat anti-mouse IgG (H + L) (Beyotime, A0428, 1:500) and DAPI were purchased from Beyotime. RPE-65 (Abcam ab231782, 1:1,000), ID3 (Cell Signaling Technology #9837, 1:500), and crystallin antibodies (Abcam, ab230722, 1:500) were used for immunofluorescence visualization (SP8; Leica).

### Tissue and Cell Processing

Adult hRPE samples were obtained from three donors (age 29–64 years old) within 24 h of death (postmortem time, 12 h). After dissecting the cornea, whole RPE tissues were collected from the remaining ocular tissue. The RPE tissues were rinsed three times with phosphate-buffered saline (PBS). First, the tissues were digested using 0.25% trypsin for approximately 30 min at 37°C until a single-cell suspension was made, and then complete Dulbecco’s Modified Eagle Mediummodified Eagle’s medium (DMEM) was used to neutralize the cell suspension. The single-cell samples were passed through a 40 μm cell strainer. Second, the cell suspension was rinsed three times with PBS and centrifuged for 5 min (1,450 rpm, 4°C). Next, a hemocytometer with trypan blue was used to count the number of cells and ensure that the level of cell viability was approximately 90%. Finally, single-cell samples were subjected to scRNA-seq analysis with a high-throughput droplet-mediated scRNA-seq platform (10× Genomics Chromium).

### Single RNA-Seq Library Construction

The appropriate volume of each sample was diluted to contain approximately 5,000 hRPE cells. Subsequently, the single-cell suspension, gel beads and oils were all added to the 10× Genomics single-cell A chip. After droplet generation, samples were transferred into 200 μL PCR tubes, and reverse transcription was performed using a T100 Thermal Cycler (Bio-Rad, California, United States). After reverse transcription, cDNA was recovered using a recovery agent provided by 10× Genomics. SPRIselect beads were used for cDNA clean-up, and then the cDNA was amplified for 10 cycles. The concentration of cDNA was detected by a Qubit2.0 fluorometer (Invitrogen, Canada). Single-cell cDNA libraries were prepared according to the Chromium Single Cell 3’ Reagent Kit v2 user guidance (https://support.10xgenomics.com/single-cell-gene-expression/index/doc/user-guide-chromium-single-cell-3-reagent-kits-user-guide-v2-chemistry).

### Real-Time PCR Analysis

We isolated total RNA using TRIzol Reagent (Ambion, Carlsbad, CA, United States). For RT-PCR, total RNA was reverse-transcribed using RT Primer Mix and oligo dT primers (MCE, China). cDNA was quantified using primers specific for mice by the ABI 7500 real-time PCR system (Applied Biosystems, Foster City, CA, United States). The sequences of the primers are provided above. PCR amplification was performed in a volume of 20 μL using RT-PCR Mix (MCE, China). The results were analyzed based on group assignment.

### Data Processing

Single cells were assessed using a 10× Chromium system (10× Genomics) and V2 single-cell reagent kits. After the library was built, the reads were aligned to the GRch38 human genome using the 10× analysis tool Cellranger Toolkit (Version 2.1.1) (Zheng et al., 2017), and a unique molecular identifier (UMI) was used to quantify gene expression. Cellranger Count was used to preprocess and preliminarily filter the data.

Then, the obtained matrix, barcode and gene files were imported into R software (version 3.6.1) for further analysis by Seurat (version 3.1.5) (Stuart et al., 2019). The cells with fewer than 200 expressed genes among the three samples were discarded. Similarly, the outliers of each sample were filtered to eliminate the signal interference of the double cells. The data were normalized using the LogNormalize method. We regressed out mitochondrial gene expression causing inherent variations, the number of UMIs per cell and even the cell cycle genes.

The FindVariableFeatures function was used to find the top 2000 highly variable genes for variable genetic parameters. Then, we used canonical correlation analysis (CCA) to correct the batch effect of the samples.

We constructed a shared nearest neighbors (SNN) diagram for the data using principal component analysis (PCA)-reduced expression data for the top 49 principal components and then used modularity optimization through the SNN clustering algorithm to identify cell clusters. We first calculated the k-nearest neighbor and performed SNN analysis; then, we optimized the modular function. Finally, the number of clusters based on the parameter resolution was determined. Next, our clusters were displayed on a 2-dimensional diagram by nonlinear dimensional reduction. We used the Seurat function RunUMAP to achieve dimension reduction. Seurat function Findmarkers were used to identify marker genes for each cluster (p.adj <0.05, avg_log2FC > 0.25).

### Cell-type-specific Transcription Factors

We used SCENIC software to select the specific transcription factors and their corresponding target genes of the hRPE clusters. Transcription factors that were active among more than 50% of cells in the macular and peripheral RPE clusters were retained by SCENIC (Aibar et al., 2017).

### Cell-Cell Interaction Analysis

iTALK (version 0.1.0) was used to analyze cell-cell interactions among hRPE clusters (Wang et al., 2019b). We used the top 50% highly expressed genes to explore the corresponding receptor-ligand pairs. The relationship between macular and peripheral RPE clusters was systematically predicted, and the top 20 pairs were displayed in the present study.

### Identification of the Cell Trajectory in hRPE Clusters

The trajectory landscape of hRPE cluster development was constructed by Monocle2 (version 2.16.0) (Trapnell et al., 2014). The highly variable genes revealed in the previous clustering examination were used to carry out pseudotime analysis. The track was then established using the DDRTree algorithm in Monocle2, and the pseudodevelopmental time was then obtained from the trajectory data.

### GO Analysis

The R package ClusterProfiler (version 3.16.0) (Yu et al., 2012) was used to perform gene enrichment analysis. The *p* value was corrected by the Benjamini & Hochberg method.

## Data Availability

The datasets presented in this study can be found in online repositories. The names of the repository/repositories and accession number(s) can be found below: GSE189770.
