# Supplementary figures and images for: A Single-Cell Transcriptome Atlas of the Human Retinal Pigment Epithelium
Source: Front Cell Dev Biol. 2021 Dec 17;9:802457. doi: 10.3389/fcell.2021.802457 (PMC8718768; doi:10.3389/fcell.2021.802457)

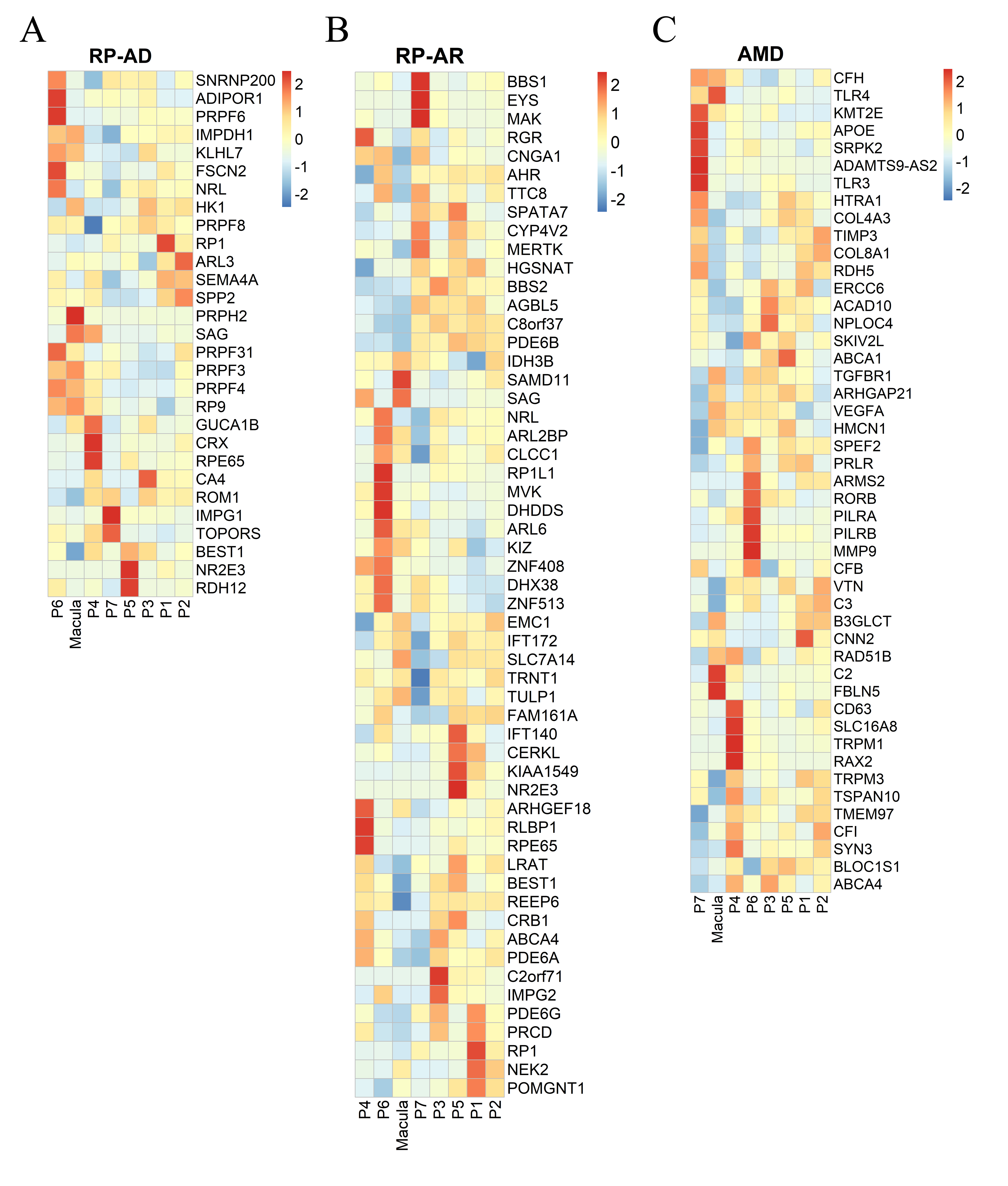

Supplement: Supplementary file 2 [file Image3.JPEG]

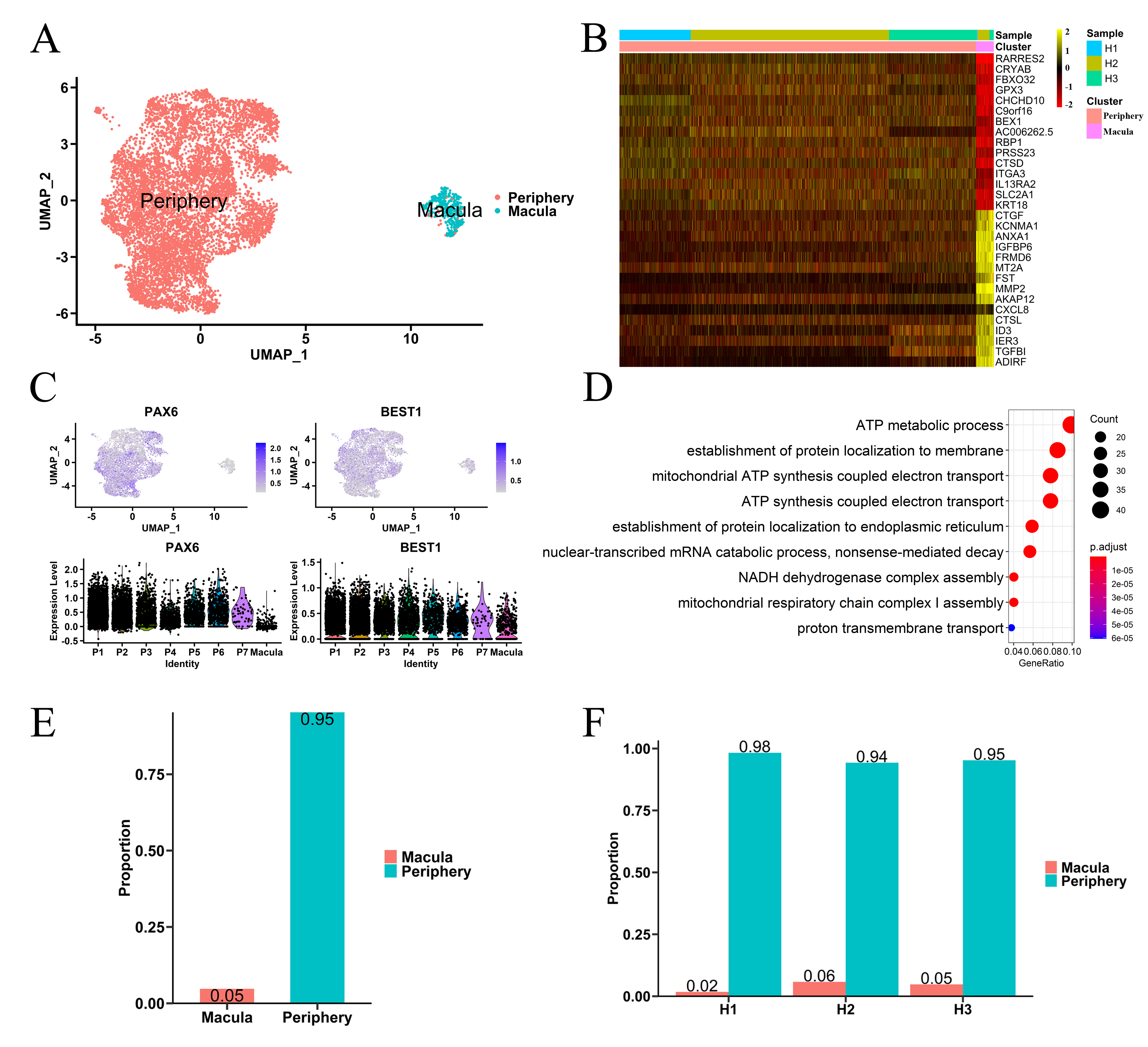

Supplement: Supplementary file 4 [file Image1.JPEG]

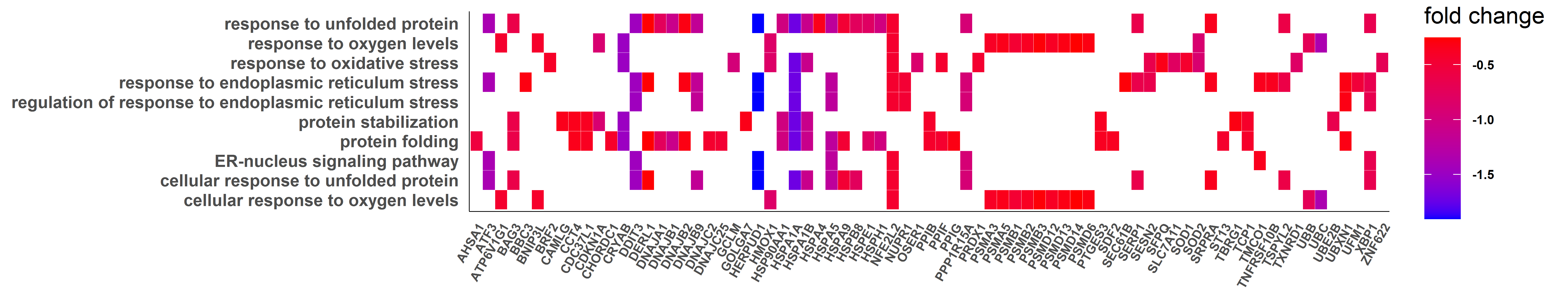

Supplement: Supplementary file 9 [file Image2.TIFF]
